# Mediating Cross-Talk: How Uterus-Derived Extracellular Vesicles Influence Early Embryo Development and Implantation

**DOI:** 10.1017/erm.2026.10034

**Published:** 2026-02-09

**Authors:** Qiuyu Yu, Lei Jin, Bo Zhang

**Affiliations:** Tongji Hospital, Tongji Medical College, Huazhong University of Science and Technology, Wuhan, China

**Keywords:** embryo–endometrium crosstalk, embryo implantation, embryonic development, extracellular vesicles, miRNA, reproductive disorders

## Abstract

**Background:**

Blastocyst formation represents an essential requirement for subsequent implantation. Successful embryo implantation depends on adequate endometrial receptivity and appropriate embryo-maternal communication. Uterus-derived extracellular vesicles (EVs), as biological nanoscale particles carrying non-coding RNAs (nc-RNAs), DNAs, proteins and lipids, play a crucial role in promoting cellular interactions and regulating maternal-foetal dialogue.

**Method:**

This article systematically searched the PubMed database and used the following keyword combinations for literature screening : (exosome * OR ‘extracellular vesicle’) AND (uter OR blastocysti) AND (blastocyst OR embryo*).

**Result:**

The composition of uterus-derived EVs exhibits variation across different physiological periods and plays different roles. Compared with the proliferative phase, EVs during the peri-implantation period contain more molecules related to cell differentiation, cell cycle, cell migration and invasion, apoptosis and antioxidant activity. The EVs discovered from uterine fluid, primary human endometrial epithelial cells (EECs), endometrial stromal cell and so forth have been shown to be internalised by embryos and trophoblast cell. The cargoes carried by EVs, mainly miRNA and proteins, regulate embryonic development and invasion-related pathways or molecules, supporting blastocyst formation and implantation. Similarly, EVs collected from dysfunctional uterus have been proved to disrupt critical reproductive processes, impairing both embryo development and implantation potential.

**Conclusion:**

This review summarises the multiple effects of uterus-derived EVs on successful embryo implantation, including the effects on pre-implantation embryo development and embryo implantation ability.

## Introduction

As nano-sized, membrane-encapsulated particles secreted from cells, extracellular vesicles (EVs) are ubiquitous in various biological fluids. They serve as important mediators of intercellular communication by encouraging the exchange of biomolecules and signal transduction. Their biogenesis primarily occurs in two ways: one is the endocytosis pathway in which the endosomes form multivesicular bodies and fuse with the plasma membrane to release the internal lumen vesicles, and the other is the plasma membrane budding pathway in which the vesicles sprout directly from the plasma membrane and fall off (Ref. 1). Based on differences in biogenesis mechanisms and physical characteristics, EVs are mainly categorised into three major subtypes: exosomes, microvesicles and apoptotic bodies. Among them, exosomes are the smallest structure with a particle size from 40 to 100 nm, apoptotic bodies are the largest with a particle size of about 1–2 μm, and microvesicles are between the two. (Ref. 2). These vesicles carry various functional molecules, including proteins, nucleic acids, lipids and mitochondrial components that can mediate the exchange of genetic information and the remodelling of phenotypes., and transport these functional ‘cargos’ to neighbouring or distant target cell to exert their function. (Ref. 3)

In recent years, the roles of EVs in maintaining organismal homeostasis, participating in immune regulation and influencing disease pathogenesis have attracted extensive attention. In the field of reproduction, EVs derived from reproductive tissues and related biological fluids are increasingly being recognised for their pivotal functions in regulating gamete maturation, the fertilisation process, embryonic development and the establishment of pregnancy (Refs 4, 5, 6).

During early mammalian pregnancy, successful embryo implantation relies on its precise coordinated interaction with the maternal uterus. Embryo implantation is strictly confined to a specific period known as the ‘implantation window’. In this critical period, the dynamic communication established between endometrium and embryo is the core mechanism ensuring the progression of embryo positioning, adhesion and invasion. The uterus not only provides essential physical support and a nutrient-rich milieu for the developing embryo but also actively participates in regulating embryonic development and implantation through the synthesis and secretion of various bioactive factors. (Ref. 7). Uterus-derived EVs as vital information-transmitting medium can regulate endometrial receptivity, influence the developmental potential of embryos and the invasive ability of trophoblast cells by carrying functional cargo such as specific miRNAs and signalling proteins (Ref. 8). Evidence suggests that cargo changes or abnormal function in uterus-derived EVs may occur in cases of implantation failure or pregnancy-related disorders compared to normal conditions, highlighting their biological significance in embryo-maternal crosstalk (Ref. 9).

**Figure 1.**
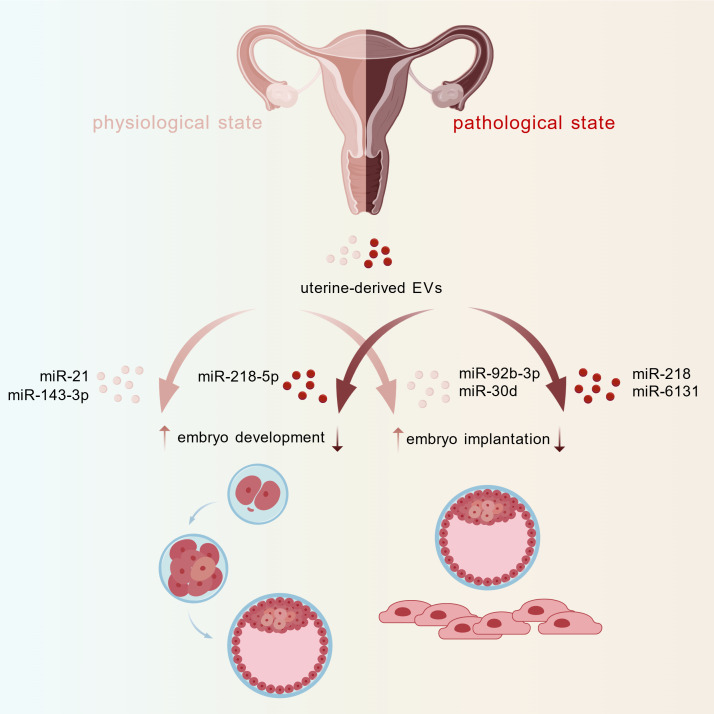
The effect of uterine-derived EVs on early embryonic development and embryo implantation with the important mi-RNA. Created with BioGDP.com.

This review will summarize the impacts of uterus-derived EVs and their cargoes on early embryonic development and embryo implantation under various physiological and pathological conditions, aiming to provide new perspectives and a theoretical foundation for related research (Figure 1).

## Result

### Regulation of uterus-derived EVs on embryonic development

In the preimplantation stage after entering the uterus from the fallopian tube, the development of the embryo is affected by the uterine microenvironment. Uterus-derived EVs, as essential components of this microenvironment, play a crucial role in regulating embryonic quality and developmental potential.

#### Effects on embryonic development mediated by uterus-derived EVs and protein cargoes

Uterus-derived EVs have demonstrated potential in promoting early embryonic development. Multiple studies confirm their overall positive effects through embryo co-culture models.

In bovine models, supplementation with luteal phase uterine EVs could significantly enhance blastocyst formation rates and hatching rates in both somatic cell nuclear transfer (SCNT) and in vitro fertilisation (IVF) embryos. This promoting effect was related to the up-regulation of IFNT the down-regulation of apoptotic and stress proteins, accompanied by increased blastocyst diameter (Refs 10, 11, 12). Sequential culture with oviductal and uterine EVs enhanced blastocyst formation rate and post-vitrification survival rate through the regulation of embryonic lipid metabolism and energy metabolism (Refs 13, 14). Porcine uterine fluid EVs collected during the early oestrous cycle also exhibited the capacity to promote trophoblast cell proliferation (Ref. 15).

Human endometrial mesenchymal stem cell-derived EVs have more profound functions: they could not only enhanced embryo quality by promoting embryonic secretion of angiogenic factors VEGF and PDGF-AA, increasing total cell count in blastocysts and improving hatching rates (Ref. 16), but also demonstrated the capacity to reverse age-related embryonic developmental decline. In mouse models, they significantly improved the declined blastocyst formation rate associated with advanced maternal age (Ref. 17). This finding suggested their potential clinical application in addressing reproductive aging outcomes. Furthermore, maternal mitochondrial DNA may be vertically transmitted to embryos through uterine fluid-derived EVs, thereby regulating embryonic energy metabolism, which provided novel insights into maternal–embryonic interactions (Ref. 18).These studies based on different sources and various animal models demonstrates the beneficial effects of uterine EVs on early embryonic development and embryo quality.

Under pathological conditions, uterine EVs exhibit negative effects. EVs acquired from the endometrial fluid of cattle with endometritis significantly lowered the blastocyst formation rate of IVF (Ref. 19). Proteomic analysis indicated that endometritis-derived EVs were rich in apoptosis and inflammation-related proteins, and these altered protein components substantially compromised embryonic development quality. The subclinical endometritis-derived EVs enriched HTRA1 protein, whose expression level is closely related to the embryonic development quality (Ref. 20).

#### Effects on embryonic development mediated by nc -RNAs cargoes

miRNAs in uterine EVs are pivotal for embryonic development as they can regulate pathways and genes related to growth and development, apoptosis and metabolism. Studies across multiple species provided direct evidence.

Thirty-seven highly expressed miRNAs including hsa-miR-26a-5p, hsa-miR-21-5p, hsa-let-7b-5p, hsa-miR-92a-3p and hsa-miR-30a-5p identified in EVs derived from human EECs were predicted to target genes enriched in core biological processes such as embryonic development, cellular metabolism, cell cycle and apoptosis (Ref. 21). When co-cultured with embryos, these EVs induced widespread changes in the blastocyst transcriptome, with 80% of these alterations being targeted by EVs miRNAs, confirming the role as key regulators of embryonic transcriptional status (Ref. 22).

Among them, the mechanism of some miRNAs had been experimentally verified. miR-21 was shown to significantly promote blastocyst formation by upregulating Bcl-2 and downregulating Bax, thereby inhibiting embryonic apoptosis (Ref. 23).This suggested that sEVs may act as a carrier of anti-apoptotic signals to embryos. let-7 was found to block trophoblast differentiation, suppress implantation, prolong blastocyst survival, and induce embryonic dormancy by inhibiting the c-myc/mTOR signalling pathway and polyamine biosynthesis (Ref. 24). This mechanism finds indirectly supported in pathological contexts. For instance: bta-let-7d was found downregulated in uterine fluid EVs from cows with endometritis.hsa-let-7a-5p and hsa-let-7f-5p were found to be enriched in EVs from adenomyosis-derived organoids during the secretory and pregnant phases, respectively. (Refs 19, 25). All these studies suggested that abnormal expression (either overexpression or underexpression) of the let-7 family may affect the intrauterine behaviour of embryos. Furthermore, miR-143-3p in porcine uterine fluid EVs directly targeted the GPD2 gene, effectively promoting trophoblast cell proliferation (Ref. 26).

Cross-species bioinformatics analysis and prediction analysis further revealed the function: miRNAs in uterine fluid EVs were significantly enriched in the core pathways of embryonic development such as Wnt, Hippo, TGF-β, FoxO and mTOR (Refs 11, 27). Bovine uterine fluid EVs miRNAs were proposed to support early embryonic development potentially through regulating lipid metabolism (Ref. 28), while human uterine fluid EVs miRNAs were predicted to target key transcription factors that regulate the specificity and function of the trophoblastic ectoderm(TE) lineage including GATA3, GATA6, SOX2, NR5A2 and TEAD1 (Ref. 29). miR-26a-5p was detected in porcine uterine fluid EVs and was predicted to regulate embryo development-related genes in trophoblast cells (Ref. 30).Specific embryo-related miRNAs such as hsa-miR-340-3p, hsa-miR-663a and hsa-miR-766-5p were detected in EVs secreted by decidualised human EECs as well (Ref. 31).

In the pathological models, altered expressed miRNA cargo in EVs primarily exerted their effects through core pathway proteins involved in embryonic development, including the inner cell mass (ICM) and TE. In samples of endometritis, a total of 52 miRNAs such as bta-let-7d and bta-miR-1 were down-regulated, while other 66 miRNAs (including bta-miR-708 and bta-miR-92b) were up-regulated. (Ref. 19).bta-miR-1 was previously showed highly expressed in uterine fluid EVs from low-fertility dairy cows, and it cooperated with bta-miR-181a to suppress mRNA expression of MAPK pathway components AP1 and IFNT in bovine conceptuses (Ref. 32). In recurrent implantation failure (RIF) patients, highly enriched miR-218-5p in uterine EVs inhibited key transcription factors of TE and ICM specification, thereby reducing blastocyst formation rate and hatching rate. This detrimental impact was reversed through the engineered EVs encapsulated with anti-miR-218-5p (Ref. 33). Adenomyosis-derived endometrial organoid EVs showed different miRNA expression profiles between secretory and pregnant phases. Eighty miRNAs (including hsa-let-7a-5p, hsa-miR-92a-3p and miR-21-5p) were enriched in the secretory phase, while during pregnancy period, the levels of 60 miRNAs (including hsa-let-7f-5p, hsa-miR-30a-5p and miR-222-3p) increased. Functional analysis linked the predicted target genes to cell proliferation, apoptosis, differentiation, epithelial–mesenchymal transition (EMT), and Hippo signalling pathways which were related to associated with adenomyosis progression and implantation failure. Among all target genes, PTEN, MDM4, PLAGL2 and CELF1 were the common targets of miRNAs from both phases, participating in endometrial receptivity and embryonic development regulation (Ref. 25). Furthermore, EVs secreted by endometrial cells under hypoxic stress carried altered miRNA cargo. After being internalised by trophoblast cells, these EVs may exert negative effects by influencing NOTCH signalling and reducing expression levels of 5-hydroxytryptamine (5-HT) and basic helix–loop–helix transcription factors (Ref. 34).

These evidences together indicate that the maternal body delivers miRNAs through EVs, and finely coordinates multiple key pathways and metabolic networks necessary for embryonic development.

### Regulation of uterus-derived EVs on embryo implantation

After the embryo develops into a blastocyst, the process of implantation is completed through positioning, adhesion and invasion. At this stage, the function of uterine EVs on the embryo in successful pregnancy shifts from influencing embryo development to regulating embryo implantation.

#### Effects on embryo implantation mediated by uterus-derived EVs and protein cargoes

A large number of studies confirmed that uterine EVs are vital instrumental in regulating embryo implantation by carrying specific functional molecules. After the internalisation by trophoblast cells, they could regulate the ability of adhesion, migration and invasion. These processes are the core cellular biological basis for successful embryo implantation. in vitro and in vivo studies had confirmed that EVs from various species and different uterine cell types significantly enhanced these trophoblast functions and provided important support for embryo implantation. (Refs 35, 36, 37). Conversely, under pathological conditions such as RIF patients, EVs derived from the uterus of RIF patients had inhibitory effects on the trophoblast functions to disrupt embryo implantation (Ref. 38).

The specific protein cargo carried by EVs may provide a molecular basis for the initial adhesion of blastocysts. Proteomic analyses had identified that EVs derived from primary human EECs were enriched with adhesion-related proteins such as ANXA2, FN1 and ITGAV (Ref. 39). Similarly, EVs isolated from decidualised or oestrogen/progesterone-treated human EECs contain multiple adhesion- and invasion-associated proteins, including IGFBP-1/7, MMP-2, LAMA5 and ITGB1 (Refs 31, 40, 41, 42). Correspondingly, proteomic profiling of EVs from hormones-treated porcine endometrial cells revealed predominant enrichment of proteins involved in VEGFA-VEGFR2, TGF-β and focal adhesion pathways, which significantly enhanced the in vitro attachment rate of hatched blastocysts (Ref. 43).

In addition to directly delivering functional proteins, uterine EVs can also reshape the functional state of trophoblast cells by activating specific signalling pathways or upregulating related proteins. Compared to other uterus-related secretory components, EVs have a stronger ability to promote embryo implantation (Ref. 40). Both receptive and decidualised endometrial cells secreted EVs that promote embryo implantation. EVs secreted by hormones-treated endometrial cells in the receptive phase activate key signalling pathways and reprogram the trophoblast proteome and phosphoproteome. These coordinated changes collectively enhance trophoblast adhesion and invasion capacity, while promoting the expansion, hatching, and implantation potential of trophoblast spheroids (Refs 36, 41, 44). Notable, the levels of adhesion-related proteins ITGA6, CXCL12 and CYR61 in the secretory group of trophoblast spheres increased after intervening EVs. It reveals that uterine EVs can also affect the transmission of information from the foetus to the mother and regulate the two-way communication between them (Ref. 44). EVs derived from decidual stromal cells stimulated the SMAD2/3 signalling pathway to upregulate N-cadherin expression, thereby facilitating trophoblast invasion (Ref. 37).

In animal models, porcine uterine fluid EVs were confirmed to up-regulate the expression of migration and adhesion-related genes such as MMP13, ITGA5 and FN1 in trophoblast cells (Ref. 45), while the MEP1B carried by EVs had been demonstrated to directly and significantly promote trophoblast proliferation and migration (Ref. 35).

#### Effects on embryo implantation mediated by nc -RNAs cargoes

During embryo implantation, miRNAs carried by EVs typically contribute to the precise regulation of successful embryo implantation by influencing trophoblast cell function or modulating the levels of adhesion molecules.

Endometrial-derived miRNAs are key signals for activating trophoblast cell function and are predicted to regulate are predicted to regulate highly relevant signalling pathways for embryo implantation (Refs 46, 47).These miRNAs were typically secreted by receptive-phase EECs and were selectively delivered to embryos or trophoblast cells via EVs. For instance, highly expressed miR-92b-3p and miR-100-5p in EVs derived from porcine and human EECs significantly promoted trophoblast proliferation, migration, and invasion by targeting TSC1/DKK3 and activating the FAK/JNK signalling pathway. The corresponding in vivo functional loss experiments (intrauterine injection of miRNA antagonists) could confirm their decisive roles in embryo implantation rates (Refs 48, 49). Similarly, during the window of implantation, EVs from human uterine fluid delivered enriched miR-30d, which was loaded into EVs by hnRNPC1. This miR-30d directly upregulated embryonic adhesion molecules such as integrin ITGB3, thereby enhancing adhesive capacity (Refs 50, 51). Porcine ssc-miR-143-3p and the long nc-RNA LNC_026212 had also been demonstrated to positively regulate trophoblast proliferation and migration in both in vitro and in vivo models by targeting GPD2 and RBP4, respectively (Refs 26, 52).

At the level of negative regulation and balance, specific miRNAs ensure implantation precision, and their dysregulation can lead to implantation failure. Porcine EVs miR-155 negatively regulated embryonic adhesion and proliferation by suppressing the expression of key genes such as β-catenin and BCL2. Further studies revealed that the endogenous retrovirus PERV was essential for maintaining normal vesicle function, as its knockdown induced aberrant upregulation of miR-155, thereby inhibiting implantation (Ref. 53). Interestingly, studies indicated that uterus-derived EVs exhibited functionally distinct effects depending on their physiological context. Porcine uterine EVs collected post-pregnancy (D12 and D15 after implantation) displayed opposite functions to those before implantation – specifically suppressing trophoblast migration and invasion (Ref. 30). This may be due to the different functional requirements of embryos before and after implantation.

Under pathological conditions, uterine fluid-derived extracellular vesicles from various sources are enriched with functionally aberrant miRNAs. Although these miRNAs target distinct signalling pathways, they ultimately act in concert to induce functional alterations in trophoblast cells. In studies involving patients with recurrent implantation failure (RIF) and LPS-induced endometritis models, miR-6131 and miR-218-5p had been demonstrated to significantly impair trophoblast cell vitality. miR-6131 was predicted to potentially modulate the PI3K-Akt and MAPK pathways (Ref. 54).miR-218-5p had been experimentally verified to inhibit trophoblast cell migration and pluripotency gene expression by downregulating sFRP2 and subsequent activating the Wnt/β-catenin pathway. (Ref. 55). Conversely, in a rat model following ovarian stimulation, uterine EVs were found to drive trophoblast cell behaviour. The enriched miR-223-3p was predicted to target genes involved in cell adhesion and EMT, such as Ankrd17, Col13a1 and Stk39. (Ref. 56).

## Discussion

This review synthesises the existing research focus on the role of EVs obtained from uterus in embryonic development and implantation. Accumulating evidence indicates that uterus-derived EVs are essential component in the uterine microenvironment. By delivering functional cargo such as proteins and miRNAs, they finely regulate gene expression and biological behaviours in target cells, thereby playing a central role in reproductive success. Current studies conducted in human, murine, bovine, ovine and porcine models have reached a consistent conclusion: uterine EVs under physiological conditions promote early embryonic development and regulate embryo implantation. The cargo composition of EVs varies with different states. EVs from the secretory phase carry a higher abundance of cargo molecules associated with positive regulatory functions when compared with the proliferative phase. Conversely, EVs derived from pathological or stress conditions exert detrimental effects.

Studies investigating the regulating function of uterus-derived EVs in early embryonic development and implantation commonly base on EVs isolated from uterine fluid, primary human EECs, EECs lines (Ishikawa, EEC-1, RL95–2 and HEC-1-A) and decidual ESCs. These EVs are functionally validated through co-culture systems with embryos, primary trophoblast cells, trophoblast cell lines and trophoblast stem cells of the corresponding specie, or trophoblast cell spheroids made of HTR-8/SVneo or JAr cells.

In the study of embryonic development, parameters such as blastocyst formation rate, blastocyst hatching rate, total cell number, ICM ratio, blastocyst apoptosis level (measured by the expression of BCL2, Tunal or Caspase3), blastocyst ROS level and post-vitrification survival rate are commonly used to evaluate developmental potential and embryo quality. Key miRNAs in this field, including miR-26a-5p, let-7, miR-21, hsa-miR-92a-3p and miR-30a, have been frequently reported in multiple studies. They are predicted to target critical genes involved in embryonic development or to be enriched in key signalling pathways. The internalisation by target cells has been shown to induce transcriptomic alterations. Regarding implantation, EVs enriched with adhesion-related proteins are internalised by trophoblast cells or embryos, triggering a series of protein and signalling pathway changes. Functional assessments of implantation capacity are usually conducted using Transwell and scratch assays, trophoblast spheroid and blastocyst adhesion assays, and uterine horn injection experiments.

However, while synthesising the existing literature, a notable limitation has emerged: the data is extremely abundant but the related functions have not yet been experimentally verified. As summarised in this review, lots of studies rely solely on high-throughput sequencing and bioinformatic predicted analyses. These studies have successfully delineated differential cargo profiles of EVs under various physiological or pathological conditions and identified meaningful biological processes including ‘cell adhesion’, ‘embryonic development’ and ‘key signalling pathways’ through predictive target gene enrichment. These findings are undoubtedly pioneering and groundbreaking and they have laid a fundamental knowledge foundation for this field. But the lack of subsequent functional experimental validation means that the biological causality of these discoveries remains largely at the hypothetical stage. More rigorous functional assays are required to substantiate these observations, thereby fully elucidating the intricate molecular dialogue between the uterus and the embryo and paving the way for novel diagnostic and therapeutic strategies for reproductive disorders.

## Abbreviations

EV: extracellular vesicles
EEC: endometrial epithelial cells
EMT: epithelial–mesenchymal transition
ESC: endometrial stromal cell
ICM: inner cell mass
RIF: recurrent implantation failure
TE: trophoblastic ectoderm
